# Phytoremediation Potential of Crop Plants in Countering Nickel Contamination in Carbonation Lime Coming from the Sugar Industry

**DOI:** 10.3390/plants9050580

**Published:** 2020-05-02

**Authors:** Arianna De Bernardi, Cristiano Casucci, Daniela Businelli, Roberto D’Amato, Gian Maria Beone, Maria Chiara Fontanella, Costantino Vischetti

**Affiliations:** 1 Department of Agricultural, Food and Environmental Sciences, Marche Polytechnic University, 60131 Ancona, Italy; c.vischetti@univpm.it; 2 Department of Agricultural, Food and Environmental Sciences, University of Perugia, 06123 Perugia, Italy; daniela.businelli@unipg.it (D.B.); roberto.damato@unipg.it (R.D.); 3 Department for Sustainable Food Process, Faculty of Agriculture, Food and Environmental Science, Università Cattolica del Sacro Cuore of Piacenza, 29212 Piacenza, Italy; gian.beone@unicatt.it (G.M.B.); mariachiara.fontanella@unicatt.it (M.C.F.)

**Keywords:** phytoremediation, phytoextraction, phytostabilisation, nickel, carbonation lime, canola, spinach, sunflower, sorghum, rhizosphere

## Abstract

The phytoremediation potential of four crop species cultivated on carbonation lime coming from the sugar industry with water-soluble nickel (Ni) exceeding the Italian legal limit of 10 µg L^−1^ was assessed. Two autumn–winter species (spinach and canola) were tested with and without the addition of bentonite in a greenhouse experiment in order to overcome prolonged unfavourable weather conditions. Two spring-summer species (sunflower and sorghum) were grown in outdoor boxes. Plant species were selected among crops of interest for phytoremediation and their rotation throughout the year enable to maintain a permanent vegetation cover. Nickel concentration in different plant tissues and the concentrations of soluble and bioavailable Ni in lime were measured. In the greenhouse study, soluble Ni decreased below the legal limit in all the tests, and the combined effect of bentonite and plants reduced Ni in lime mainly in the bioavailable fraction. Spinach and sunflower emerged to be more suitable for phytoextraction than canola and sorghum, because of the higher concentration of the metal in the epigeal portions. The results from the outdoor experiment highlighted that sorghum has a good phytostabilisation potential since its ability to accumulate Ni mainly at the root level and to attract a significant amount of bioavailable Ni in the rhizosphere. This study arose from a real scenario of environmental contamination and investigated the potential of different approaches on the bioremediation of a specific industrial waste product.

## 1. Introduction

Environmental pollution by Potentially Toxic Elements (PTE) has become a serious problem since the 1940s, when rapid industrialisation and urbanisation started to cause contamination [1]. PTE cannot be biodegraded and therefore accumulate in the ecosystem and enter the food chain [2,3,4].

Several physical and chemical methods have been used for removing potentially toxic elements from contaminated matrices [5]; nowadays, these traditional remediation technologies are being replaced by biological ones, usually known as bioremediation technologies [6,7]. Compared with conventional clean-up methods, bioremediation has lower capital costs, and it is considered to be aesthetically pleasing [8]. Among bioremediation technologies, phytoremediation exploits plants to remove pollutants from the environment, or to render them harmless by degradation or immobilisation [9,10,11,12].

Since the nineties [13,14], a growing scientific and commercial interest has developed regarding this promising technology as a more eco-friendly, non-intrusive and cost-effective remediation method for the reclamation of polluted sites [15,16]. However, some limitations should be taken into account when applying phytoremediation: long-term efficiency, the depth of action limited to the root zone, the need for proper handling and disposal of the biomass produced and a careful evaluation of the specific site scenario (i.e., plant requirements—pollution level) [17,18,19,20]. Depending on the nature of the pollutants and their concentration, the choice for the optimal species is a function of the encroachment capability given by tolerance mechanisms (exclusion and detoxification) that allow for maintaining a low concentration of the potentially toxic element in the cytoplasm and the most sensitive compartments of plant cells [21]. Likewise, information regarding the substrate and climate of the place where the remediation is requested, and the site accessibility for the cultivation machines [22] should be compared with the needs of the crop to be used (i.e., temperatures required in the development phases, water and nutritional needs) [23]. In more detail, substrate aeration, pH, cation exchange capacity (CEC) and the presence of organic matter, clay components and the elements available affects the bioavailability of potentially toxic element and thus their accumulation in plant tissues [24,25,26,27].

According to the different mechanisms involved, there are several phytoremediation approaches for the PTE decontamination of solid matrices: phytoextraction (pollutants taken up into the plant biomass), phytostabilisation (limiting the mobility and bioavailability in soil by plant roots), and phytovolatilisation (conversion of pollutants to a volatile form and their subsequent release into the atmosphere) [28,29]. Depending on the phytoremediation method, the specific features of many plant species can prove useful [30]. Usually, hyperaccumulator plants that can accumulate high amounts of PTE in their above-ground tissues without adverse effects [14,31,32,33] are chosen for phytoextraction, but such plants typically produce small amounts of biomass and have no economic value [34]. The species used for phytostabilisation must grow rapidly with a well-developed root system but do not need particular habitat requirements [35]. The translocation factor (TF) given by the ratio between the concentration of PTE in the aerial part of the plants and the concentration of the same in the roots is one of the key factors in the evaluation of plants for phytoextraction processes [36]. A TF higher than 1 signifies tolerance of the plant towards the PTE and its ability to move it in the epigean portions, and, according to the Baker theory, this plant is considered a good accumulator usable for phytoextraction [37,38].

Combining two or three different remediation approaches can lead to more effective results [39]. Aided phytostabilisation [40], or chemophytostabilisation [41], is an interesting example of a co-remediation technique in which amendments such as clay minerals, organic compost, phosphates, lime and zero-valent iron are applied in the polluted substrate to help the plants to deactivate or immobilise PTE [42,43,44,45].

Among PTE, mercury (Hg), copper (Cu), silver (Ag), cadmium (Cd), zinc (Zn), lead (Pb), chrome (Cr), cobalt (Cb) and nickel (Ni) are considered the most toxic [46], but all elements in high concentrations are potentially harmful [47]. The sources of PTE in the environment may be natural (geogenic origin) or anthropogenic from activities such as mining, burning of fossil fuel, fertiliser application, disposal of household debris, municipal and industrial wastes [48,49]. As described by Spellman [50], certain recyclable wastes like biosolids from sewage treatment plants, food processing companies, and other sources are commonly reused as fertilisers. However, some careful testing is needed in order to control problems concerning the eventual release of water-soluble, mobile pollutants.

This research came from a collaboration with Sadam Spa, an Italian company in the sugar industry, involved in an environmental recovery project of a disused quarry. According to the Italian Legislative Decree n.186 [51], the recovery project was aimed at remodelling the morphology of the quarry using lime from sugar refining as a filling material. The subsequent assessment of chemical pollutants in lime, conducted by Marche Regional Environmental Protection Agency (ARPAM), highlighted a contaminated area exceeding the legal limit of soluble Ni concentration (10 μg L^−1^).

The aim of this study was to assess the phytoremediation capacity of four plant species—spinach (*Spinacia oleracea*) and canola (*Brassica napus*) in a greenhouse study, and sorghum (*Sorghum vulgare*) and sunflower (*Helianthus annuus*) in an outdoor experiment—in order to decrease the concentration of the soluble Ni below the legal limit set by the Italian Legislative Decree n.186 [51]. This study was performed to support the application of phytoremediation techniques required for the remediation of a real contaminated site. Crop plants have most of the properties required by real-case applications of phytoremediation techniques, such as wide availability, cheapness, ease of growing and consistent biomass production [52,53,54]. Nevertheless, since the ability to tolerate and accumulate potentially toxic element is also a fundamental requirement, crop species already known in the scientific field for their metal storage capacity have been selected [10,23,55,56,57,58,59,60,61,62,63,64,65,66]. Moreover, as these species have either an autumn–winter cycle (canola and spinach) or a spring–summer cycle (sorghum and sunflower), their rotation throughout the year permit to maintain a permanent vegetation cover in the contaminated site.

Aided phytoremediation was tested using bentonite in the greenhouse experiment since a previous laboratory study on the same substrate showed that this clay mineral is a good sequestrant of Ni when it is added at doses of 5% (*w*/*w*) [67].

The results highlight that spinach and sunflower should be preferred for phytoextraction over canola and sorghum, given their ability to store Ni in the epigeal portions. In the greenhouse study, soluble Ni decreased below the legal limit in all the tests, and the combined effect of bentonite and plants reduced Ni in lime primarily in the bioavailable fraction. İn the outdoor experiment, the metal was accumulated mainly in sorghum roots, and a significant amount of bioavailable Ni was found in the rhizosphere, indicating sorghum as a good option for the phytostabilisation technique.

## 2. Results

### 2.1. Greenhouse Experiment

#### 2.1.1. Nickel in Lime

**Figure 1:**
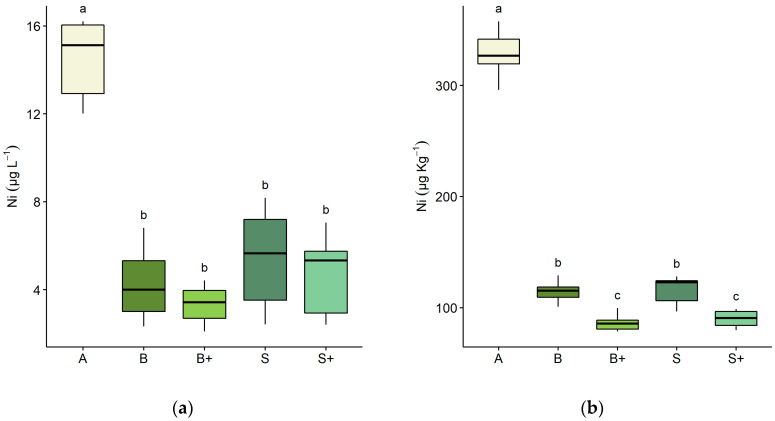
Concentration of nickel in lime before and after plant harvest. (**a**) Soluble Ni (µg L^−1^) in lime samples; (**b**) bioavailable Ni (µg Kg^−1^) in lime samples. Letters on the x-axis refer to concentration before sowing (A) and after the harvest of: *Brassica napus* without bentonite (B) and with bentonite (B+); *Spinacia oleracea* without bentonite (S) and with bentonite (S+). Lower case letters refer to Dunn’s Kruskal–Wallis multiple comparisons (Benjamini–Hochberg *p*-value adjustment, α-level = 0.05).

Figure 1 reports the concentration of soluble and bioavailable nickel in lime before and after the plant harvest. Before sowing, Ni concentration was similar among samples since the inter quartile range values were 3.0 (median 15.0) and 21.7 (median 331.0) for soluble and bioavailable fractions, respectively. A considerable decrease in soluble Ni concentration was observed following the harvest compared to the initial concentration (Figure 1a), and differences between trials were not significant, even when bentonite was added to contaminated lime. Bioavailable Ni was significantly reduced in all treatments (Figure 1b). Regardless of the species, the addition of bentonite further decreased bioavailable Ni in lime. The median concentration of Ni after treatments was slightly lower using canola compared to spinach, both in soluble and bioavailable fractions (Figure 1).

#### 2.1.2. Nickel in Plants

**Figure 2:**
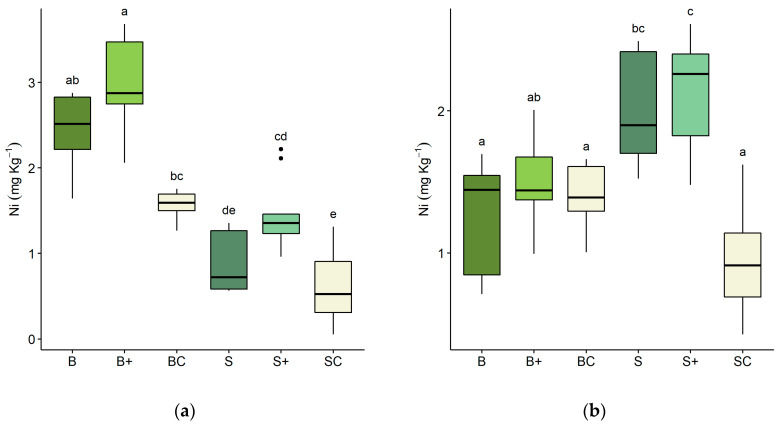
Nickel concentration (mg kg^−1^ dry weight) in plants. (**a**) Hypogeal portion; (**b**) epigeal portion. Letters on the x-axis refer to concentration in: (B) *Brassica napus* plants grown on contaminated lime without bentonite (B), with bentonite (B+) and *Brassica napus* plants grown on uncontaminated lime as a control (BC); *Spinacia oleracea* plants grown on contaminated lime without bentonite (S) and with bentonite (S+) and *Spinacia oleracea* plants grown on uncontaminated lime as a control (SC). Lower case letters refer to Dunn’s Kruskal–Wallis multiple comparisons (Benjamini–Hochberg *p*-value adjustment, α-level = 0.05).

Figure 2 reports nickel concentrations in plants. Ni concentration in roots (Figure 2a) was significantly higher in canola than in spinach, and, for both species, the addition of bentonite led to a higher accumulation of the metal that differed significantly from the control. Concerning the epigeal portion (Figure 2b), Ni accumulation was significantly higher in spinach than in canola and considerably higher than the control, while, in canola, no significant differences were found between treated and control plants. The addition of bentonite does not seem to significantly affect metal accumulation in either hypogeal or epigeal portions.

The translocation factor of the plants grown in the greenhouse experiment, calculated as the ratio between the concentration of Ni in epigeal and hypogeal tissues, is shown in Table 1. Between the two tested species, spinach is the one that presents in all trials a translocation factor higher than one.

The biomass production of plants grown on contaminated lime was similar to that measured for control plants within the same species (Table S1); in particular, the average weights of the hypogeal and epigeal portions of canola plants were 4.48 ± 0.75 and 14.97 ± 1.16 g, respectively, while, for spinach plants, these weights were 0.57 ± 0.21 and 5.54 ± 0.74 g, respectively. Considering the metal concentrations in the various plant tissues and the biomass produced, the mean values of phytoextracted Ni per plant were 30.9 ± 6.4 (B), 34.3 ± 4.6 (B+), 28.3 ± 3.5 (BC), 12.5 ± 0.8 (S), 10.7 ± 2.1 (S+) and 5.9 ± 0.2 µg (SC).

### 2.2. Outdoor Experiment

#### 2.2.1. Nickel in Lime

**Figure 3:**
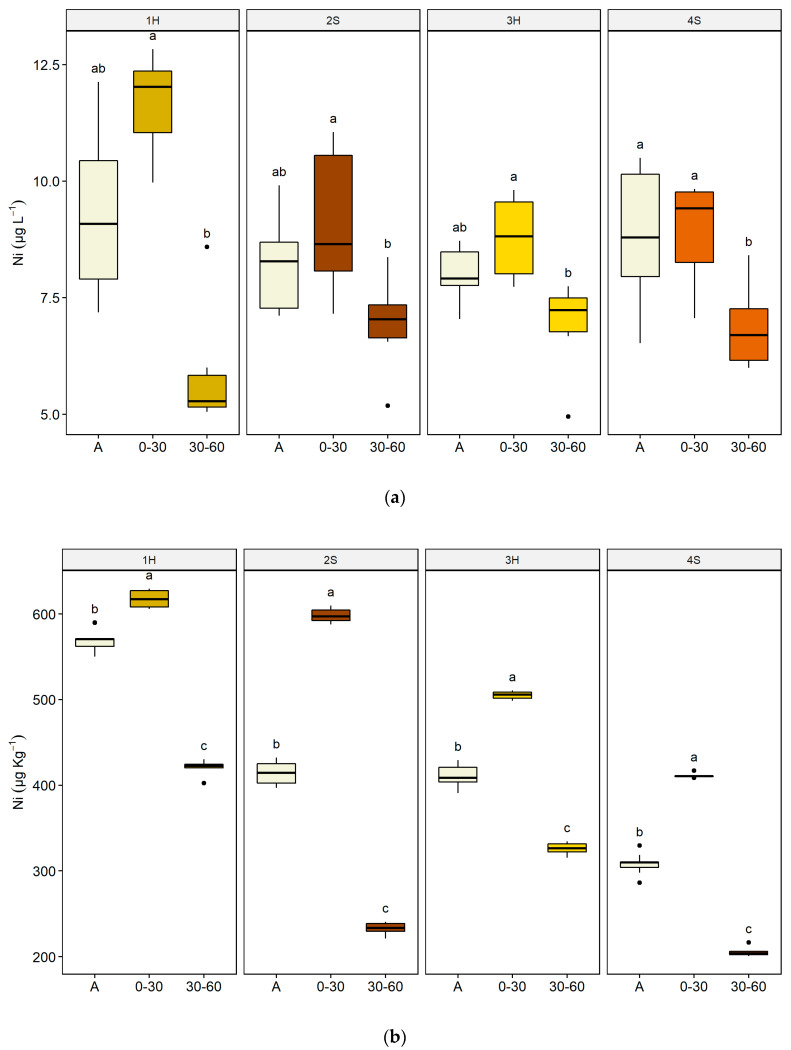
Concentration of nickel in lime in boxes 1–4 before and after plant harvest. (**a**) Soluble Ni (µg L^−1^); (**b**) bioavailable Ni (µg Kg^−1^). Codes on the x-axis refer to initial concentration (A), and concentration after the plant harvest grouped by sampling depth (0–30 cm, 30–60 cm). Boxes 1 and 3 cultivated with *Helianthus annuus* (1H; 3H); boxes 2 and 4 cultivated with *Sorghum vulgare* (2S; 4S). Lower case letters refer to Multiple comparisons in each box (Dunn’s Kruskal–Wallis, Hochberg *p*-value adjustment, α-level = 0.05).

Figure 3 reports the concentration of soluble and bioavailable nickel in the four boxes used for the outdoor experiment, before and after the plant harvest.

The initial concentration of soluble Ni was similar for the four boxes, with medians varying from 7.9 µg L^−1^ (Box 3) to 9.1 µg L^−1^ (Box 1) and no significant differences were highlighted using the Kruskal–Wallis test (*p* > 0.05) (Figure S1a). On the contrary, regarding the initial concentration of bioavailable Ni, box 1 had the highest median (570.7 µg Kg^−1^), while box 4 had the lowest (309.9 µg Kg^−1^), and significant pairwise differences (Dunn’s test) were found (Figure S1b). Boxes 2 and 3 were the only ones with similar initial soluble and bioavailable Ni concentrations (Figure S1).

A similar Ni distribution pattern between pre- and post-harvest concentrations was observed in all boxes (Figure 3). After the harvest, Ni concentrations were significantly higher at 0–30 cm of depth respect to 30–60 cm of depth for both fractions, and this behaviour was particularly evident for the bioavailable Ni (Figure 3b).

After the harvest, a comparison of the boxes showed no significant differences in soluble and bioavailable Ni for box 2 cultivated with sorghum and box 3 cultivated with sunflower, at both sampling depths (Figure S2).

Table 2 shows the concentration of soluble and bioavailable Ni in the rhizosphere of the plants at the end of the experiment. No significant differences between plants and boxes were found for soluble Ni, while the bioavailable fraction was high in all cases—the highest being for sorghum in box 4. A lower pH value (7.84 ± 0.04) was measured on the rhizosphere samples with respect to all the other samples.

#### 2.2.2. Nickel in Plants

**Figure 4:**
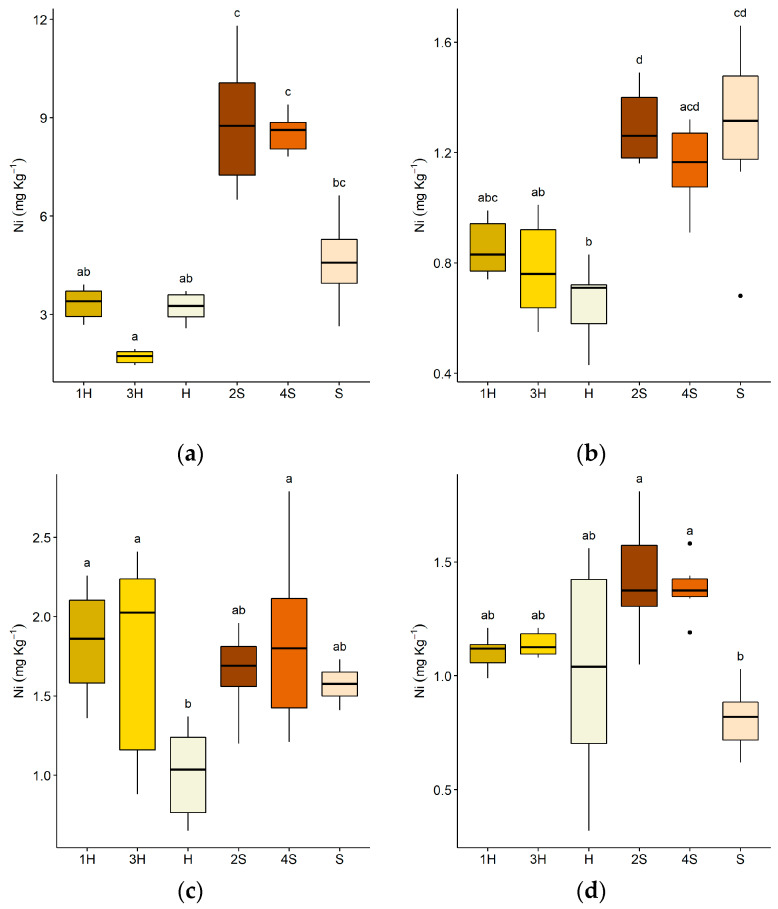
Ni concentration (mg kg^−1^ dry weight) in the different portions of the plants. (**a**) Roots, (**b**) stems, (**c**) leaves, (**d**) infructescences. Codes on the x-axis refer to concentration in: (1H) *Helianthus* in box 1; (3H) *Helianthus* in box 3; (H) *Helianthus* control in uncontaminated lime; (2S) *Sorghum* in box 2; (4S) *Sorghum* in box 4; (S) *Sorghum* control in uncontaminated lime. Lower case letters refer to Dunn’s Kruskal–Wallis multiple comparisons (Benjamini–Hochberg p-value adjustment, α-level = 0.05).

Figure 4 shows the Ni accumulation in different parts of the plants grown either in the four boxes or in the uncontaminated substrate. Ni was accumulated more in the roots than in the other parts by both plants species (Figure 4a). In roots and stems, Ni accumulation was higher in sorghum than in sunflower (Figure 4a,b). Sorghum roots accumulated a higher concentration of Ni than the control, even if the differences are not statistically significant, while this was not true for sunflower plants and for both species in stems. Similar Ni concentrations were found in leaves and infructescences, regardless of the plant species (Figure 4c,d). Significant differences with respect to the controls were found for sunflower in leaves and for sorghum in infructescences.

The translocation factor regarding the plants in the outdoor experiment is shown in Table 3. In contrast to sorghum, sunflowers grown in both boxes 1 and 3 showed a TF greater than one.

The biomass produced by both sorghum and sunflower plants grown in the contaminated boxes was similar to that measured in the respective controls (Table S2). The average dry plant weights (g) recorded for roots, stems, leaves and infructescences were 25.55 ± 3.31, 85.75 ± 4.86, 37.66 ± 8.44 and 35.86 ± 3.25 for sunflower, and 15.74 ± 2.16, 38.57 ± 2.34, 21.37 ± 2.38 and 14.16 ± 2.64g for sorghum, respectively. The mean values of phytoextracted Ni per plant were as follows: 230.2 ± 11.3 (1H), 234.9 ± 21.4 (3H), 223.2 ± 9.3 (H), 231.1 ± 27.0 (2S), 246.3 ± 10.2 (4S) and 165.3 ± 27.8 µg (S).

## 3. Discussion

### 3.1. Greenhouse Experiment

In the greenhouse experiment, the phytoremediation capacity of canola (*Brassica napus*) and spinach (*Spinacia oleracea*) was tested with and without the addition of 5% of bentonite.

Many authors have studied the phytoextraction capacity of *Brassica napus* [68,69,70] and *Spinacia oleracea* [71,72,73] and the effect of the addition of sequestrants such as bentonite in decreasing the available form of potentially toxic element [67,74,75,76].

In the present experiment, the Ni soluble fraction following the harvest was considerably reduced, and a concentration under the legal limit of 10 μg L^−1^ was achieved in all trials (Figure 1a).

Canola showed a higher concentration of the metal in the hypogeal portion than in the epigeal portion, and similar patterns of Ni accumulation have been found in previous studies [47,77]. In agreement with other authors [71,72,78,79], the opposite behaviour was observed for spinach, which accumulated Ni mostly in the aerial tissues.

As highlighted by the results, spinach seems suitable for the phytoextraction of Ni in lime, while the low translocation factor of canola makes it appropriate for phytostabilisation techniques. With a view to a practical application of phytoremediation techniques, the differences in biomass production between species should be taken into account, as the biomass of canola is considerably higher than that of spinach, as reported above.

The addition of bentonite may improve phytoremediation outcomes since it reduces both the soluble and bioavailable fractions of Ni due to the capacity of this mineral to adsorb the metal in its external and internal sites, as described by other authors [75,80,81]. As reported in previous work [67], the competition of bentonite adsorption sites with Ni adsorbed on organic matter and the stabilisation of the metal in internal adsorption sites of bentonite could reduce the available concentration of Ni in lime (Figure 1b). The above findings support the use of a combination of sequestrants and plants in a practical case of bioremediation.

### 3.2. Outdoor Experiment

Before sowing, the concentrations of soluble and bioavailable Ni measured in the four boxes were different with respect to the values observed for lime collected in a previous field survey and used for the greenhouse experiment. This different starting condition was not surprising, considering that six months had elapsed between the two sampling campaigns, during which the site was subject to weather events, speciations and mobilizations of the metal may have occurred. Nevertheless, the low soluble Ni concentrations detected in May 2018 provided a real case for a typical phytoremediation trial, since high concentrations and lack of time represent the main limitations for the application of this technique [82].

After harvesting, there was a clear tendency of the soluble and available forms of the metal to move towards the first 0–30 cm in depth both in the sunflower and the sorghum boxes (Figure 3); this depth represents the layer where the root system is developed, indicating that the metal distribution in the boxes could be due to the attraction exerted by the roots and by their radical exudates. The function of roots in attracting and sequestering potentially toxic element has been highlighted by many authors who all agree that the presence of metals induces a higher production of root exudates, such as low molecular weight organic acids [83,84,85], which play an important role in the immobilisation of the available form of metals.

Boxes 2 and 3, which had similar initial Ni concentrations, showed no significant differences in the levels of soluble and bioavailable Ni measured after the harvest of sorghum and sunflower, respectively (Figure S1). Despite these results, in sorghum stems and root tissues, the Ni concentration was significantly higher than in sunflower plants (Figure 4a,b), but at the end of the trials, this difference in extraction capacity was compensated by greater production of biomass by the sunflower compared to the sorghum.

The noticeable capability of sorghum to accumulate metals in roots was also reported in other studies [86,87,88] in which the authors suggest to use sorghum spp. in phytostabilisation rather than in phytoextraction experiments.

A further demonstration of the ability of root apparatus to attract the available form of Ni is reported in Table 2, where the concentration of bioavailable Ni in the rhizosphere of the plants always proved to be higher than in the 0–30 cm layer after harvest.

Although sunflower plants accumulated a considerable amount of Ni in the root portion, their translocation factor showed values higher than 1; therefore, these plants could be used for phytoextraction purposes on this lime. Finally, the presence of a high content of organic matter (OM) in the substrate (Table 4) might have helped in metal sequestration, because OM promotes the formation of OM-Ni complexes and exudate production by the roots of the plants [84].

## 4. Materials and Methods

The experimental design was performed with two different case studies: a greenhouse experiment with autumn–winter plants and an outdoor experiment with summer–spring plants.

In both tests, the substrate used to grow the plants was carbonation lime, a residue of the industrial process of sugar juice purification, made up of 90% calcium carbonate [67]. This substrate was sampled where the lime was used as the filling material for a disused quarry in a recovery project (municipality of Monte Roberto, Marche, Italy). The samples were collected from four square plots (2 m side) equally spaced along a transect covering the whole length of the contaminated area mapped by ARPAM. The main properties of the substrate were determined from samples collected during two different field surveys, one in autumn 2017, and one in spring 2018. A clearly alkaline reaction and a high organic matter content were found (Table 4). The substrate pH was determined by a glass electrode in distilled water (pH H_2_O) suspensions at a 1:2.5 soil to liquid ratio. Total Ni concentration was determined by the acid digestion method; the dry and 2-mm sieved substrates were digested in HNO_3_, 65% v/v, (28 L/Kg) overnight, 2 mL of H_2_O_2_, 30% v/v, was then added, and, after 6 h, the blend was heated to 90 °C for 90 min and filtered [87]. Analyses of the eluates were performed using an ICP-OES (Inductively Coupled Plasma—Optical Emission Spectrometer, Agilent 5100 VDV). The organic matter was determined according to Walkley and Black [89].

An uncontaminated carbonation lime (soluble Ni concentration under 2 µg L^−1^), collected from the same site but outside of the contaminated area, was used to cultivate plants as a reference control.

### 4.1. Greenhouse Experiment

Because of prolonged unfavourable weather conditions that occurred at the contaminated site during autumn–winter months, the experiment was conducted in the experimental greenhouse at Marche Polytechnic University, Ancona, Italy, with a daytime temperature of 24 ± 3 °C and a night temperature of 20 ± 3 °C. Plants of *Spinacia oleracea L.* (spinach) and *Brassica napus L.* (canola) were grown separately in plastic jars having a volume of 3 litres. The plants were grown under natural photoperiod and with a relative humidity of 60% ± 2%.

Lime samples were collected from each of the four square plots delineated in the contaminated area in November 2017, and they were mixed carefully, air-dried and 2-mm sieved. After processing, the concentrations of soluble and bioavailable Ni in nine sub-samples were measured to ensure that both Ni fractions were homogeneously distributed in the substrate.

Six trials were set up in eighteen jars (three replicates per trial) as follows: *Brassica* without bentonite (B), *Brassica* with bentonite added at 5% (*w*/*w*) (B+), *Brassica* control (BC), *Spinacia* without bentonite (S), *Spinacia* with bentonite added at 5% (*w*/*w*) (S+) and *Spinacia* control (SC). Commercial activated bentonite with a mesh size of 100–500 µm, composed of 85%–90% montmorillonite and 1%–4% inert silica was used. The main physico-chemical characteristics of the bentonite used were: a surface area of 88.0 m^2^ g^−1^; a CEC of 150 meq/100 g; and a pH of 9.5 (sol. 5%).

To prevent emergence failures, ten seeds were initially sown in each jar. Subsequently, only four seedlings per pot for canola and six for spinach were allowed to grow.

Manual irrigation was provided to avoid water stress from the time of sowing up to the harvesting at the end of the vegetative cycle of the crops (approximately 110 days).

After the harvest, all the plants from each jar were sampled, gently washed with deionised water, separated into the epigeal and hypogeal portion, oven-dried (70 °C until the plant tissue was completely dry), weighed, milled and analysed to assess the concentration of Ni in the tissues.

At the end of the experiment, three samples of substrate were collected from each jar, obtaining nine replicates per trial. After re-drying, the concentration of soluble and bioavailable fraction of Ni was measured.

### 4.2. Outdoor Experiment

Lime samples were collected from each of the four square plots delineated in the contaminated area in May 2018. Four boxes were placed directly in the study area, exposed to weather conditions, and each was filled with samples coming from a different square plot, in order to represent the spatial heterogeneity of the Ni distribution in the site.

Nine sub-samples were collected from each 1 m^3^ box, along independent zig-zag paths at different depths to achieve randomness and to measure the soluble and bioavailable Ni before sowing.

Boxes 1 and 3 were sown with *Helianthus annuus L.* (sunflower), while boxes 2 and 4 were cultivated with *Sorghum vulgare L*. Only nine sunflower plants and fifteen plants of sorghum were allowed to grow per box. Single manual irrigation was provided immediately after sowing.

At the end of the biological cycle (around 90 days), a third of the plants grown in each box were harvested whole and then carefully separated into roots, stems, leaves and infructescences, oven-dried (70 °C until the plant tissue were completely dry), weighed, milled and analysed to determine the Ni contents.

After plant eradication, both the substrate intimately adhering to the root surface (rhizosphere) and the lime from each box at two different depths (0–30 and 30–60 cm) were collected in order to assess the distribution of soluble and bioavailable Ni fractions following cultivation.

### 4.3. Ni Extraction and Analysis

#### 4.3.1. Substrate

The soluble Ni extraction was carried out with distilled water (1 g/10 mL) according to the cession-test reported in the Italian Legislative Decree n. 186 [90]. The duly sealed samples were subjected to agitation and the liquid/solid separation was then reached by centrifugation for 5 min and filtering.

The bioavailable fraction was extracted with a solution of Diethylenetriaminepentaacetic acid (DTPA), CaCl_2_ · 2H_2_O (0.01 M) and triethanolamine (0.1 M) at pH 7.3 (1 g/2 mL), following the indications in the Italian Official Gazette n. 248 [91].

Analyses of the eluates were performed using a plasma emission spectrometer ICP-OES Agilent 5100 VDV. The operating analysis of the system was as follows: radio frequency power 1.4 kW, plasma gas flow 12 L min^−1^, auxiliary gas flow 1.0 L/min, nebulizer flow 0.7 L min^−1^, observation view axial, replicate readings 3, selected Ni wavelengths 231.604 nm.

#### 4.3.2. Plants

The dry milled plant tissues were digested in HNO_3_ (28 L/Kg) overnight, 2 mL of H_2_O_2_ was then added (30%), and, after 6 h, the blend was heated to 90 °C for 90 min and filtered [92].

Analyses of the eluates were performed using a plasma emission spectrometer ICP-OES Agilent 5100 VDV. The operating analysis of the system was as follows: radio frequency power 1.4 kW, plasma gas flow 12 L min^−1^, auxiliary gas flow 1.0 L/min, nebulizer flow 0.7 L min^−1^, observation view axial, replicate readings 3, selected Ni wavelengths 231.604 nm.

### 4.4. Statistical Analysis

Nonparametric tests were used because of non-normal distributions. The significance of the differences in the content of Ni in different morphological parts of the plants and in lime samples was calculated using the Kruskal–Wallis test and Dunn’s post-hoc test. Either Benjamini–Hochberg or Hochberg *p*-value adjustments were used depending on the number of tests performed, as suggested in literature [93,94]. Statistical analyses were performed in R software (R Development Core Team 2018 version 3.5.2) using ‘dunn.test’ package version 1.3.5.

## 5. Conclusions

Spinach and sunflower emerged to be suitable for the phytoextraction of Ni in lime, while the low translocation factor of canola and sorghum makes them more appropriate for phytostabilisation techniques. The addition of bentonite may improve phytoremediation outcomes since this mineral reduces both the soluble and, to a greater extent, the bioavailable fractions of Ni due to its capacity to adsorb the metal. The above findings support the use of a combination of sequestrants and plants in a practical case of bioremediation.

The results from the outdoor experiment highlight that sorghum has a good phytostabilisation potential due to its ability to accumulate Ni mainly at the root level and to attract a significant amount of bioavailable Ni in the rhizosphere. Moreover, the response of plants to the presence of potentially toxic elements may vary according to the elements and the different concentrations of their available forms.

For both experiments, limited differences in biomass production between plants grown on contaminated lime and those grown on control were observed, this is probably due to the Ni concentrations in the tissues, which were in all cases lower than the phytotoxicity threshold of 50 mg Kg^−1^ indicated for moderately tolerant species to this metal [95].

The focus of future research could take in account several unresolved issues concerning the use of bioremediation for sites contaminated with PTE, studying the possibility of using plants that are even more efficient in the hyper-accumulation of metals and are able to develop a wide and deep root apparatus. Studies on the possibility to solve practical problems of contamination with high metal concentrations should be performed, combining them with the use of different sequestering minerals that are able to immobilise the metal in the plant root layers. Other interesting proposals to enhance the phytoremediation performance could be the use of plant growth-promoting bacteria (PGPB) and melatonin, which biostimulate the plant growth and improve the plant tolerance against PTE [96,97,98].

## Figures and Tables

**Table 1:** Translocation factor for Ni in plants growing in the greenhouse experiment (reported values are the medians of nine replicates and interquartile range).

Trial	TF
B	0.57 (0.14)
B+	0.51 (0.12)
BC	0.83 (0.07)
S	2.70 (0.99)
S+	1.67 (0.70)
SC	1.52 (0.54)

**Table 2:** Soluble (µg L^−1^) and bioavailable (µg Kg^−1^) Ni concentration in the rhizosphere (reported values are the median of nine replicates and interquartile range). Different letters indicate significant differences (ns: not significant differences), Dunn’s Kruskal–Wallis multiple comparisons test (Benjamini–Hochberg p-value adjustment, α-level = 0.05).

Box	Plant	Soluble Ni	Bioavailable Ni
1	*Helianthus annuus*	10.2 (0.7) ^ns^	681.2 (74.4) ^ab^
2	*Sorghum vulgare*	8.0 (0.6) ^ns^	637.9 (10.3) ^a^
3	*Helianthus annuus*	10.3 (1.7) ^ns^	692.2 (78.9) ^ab^
4	*Sorghum vulgare*	8.4 (1.5) ^ns^	763.1 (15.7) ^b^

**Table 3:** Translocation factor for Ni in plants growing in the outdoor experiment (reported values are the medians of six replicates and interquartile range).

Box	TF
1H	1.07 (0.26)
3H	2.04 (0.30)
H	0.84 (0.23)
2S	0.52 (0.13)
4S	0.51 (0.05)
S	0.83 (0.31)

**Table 4:** Basic properties of the substrate used for the experiments.

Parameter	Values
pH	8.36 ± 0.10 ^1^
Organic matter (%)	7.2
Total Ni (mg Kg^−1^)	2.62 ± 0.63 ^1^

^1^ Reported pH and Ni concentrations are means of sixteen replicates ± standard deviation.

## Supplementary Materials

The following are available online at https://www.mdpi.com/2223-7747/9/5/580/s1, Figure S1: Pre-sowing concentration of soluble (a) and bioavailable (b) Ni in lime in boxes 1–4. Figure S2: Post-harvest concentration of soluble (a) and bioavailable (b) Ni in lime in boxes 1–4. Table S1: Biomass production of plants in the greenhouse experiment. Table S2: Biomass production of plants in the outdoor experiment.

## References

[1] Merian E. (1984). Introduction on environmental chemistry and global cycles of chromium, nickel, cobalt beryllium, arsenic, cadmium and selenium, and their derivatives†. Toxicol. Environ. Chem..

[2] Bian R., Joseph S., Cui L., Pan G., Li L., Liu X., Zhang A., Rutlidge H., Wong S., Chia C. (2014). A three-year experiment confirms continuous immobilization of cadmium and lead in contaminated paddy field with biochar amendment. J. Hazard. Mater..

[3] Lv S.W., Liu J.M., Wang Z.H., Ma H., Li C.Y., Zhao N., Wang S. (2019). Recent advances on porous organic frameworks for the adsorptive removal of hazardous materials. J. Environ. Sci. (China).

[4] Ali H., Khan E., Ilahi I. (2019). Environmental chemistry and ecotoxicology of hazardous heavy metals: Environmental persistence, toxicity, and bioaccumulation. J. Chem..

[5] Brusseau M.L., Pepper I.L., Gerba C. (2019). Environmental and Pollution Science.

[6] Rodríguez-Eugenio N., McLaughlin M., Pennock D. (2018). Soil Pollution: A hidden Reality.

[7] Sharma S., Tiwari S., Hasan A., Saxena V., Pandey L.M. (2018). Recent advances in conventional and contemporary methods for remediation of heavy metal-contaminated soils. 3 Biotech.

[8] Vidali M. (2009). Bioremediation—An overview. Pure Appl. Chem..

[9] Raskin I., Smith R.D., Salt D.E. (1997). Phytoremediation of metals: Using plants to remove pollutants from the environment. Curr. Opin. Biotechnol..

[10] Salt D.E., Blaylock M., Kumar N.P., Dushenkov V., Ensley B.D., Chet I., Raskin I. (1995). Phytoremediation: A novel strategy for the removal of toxic metals from the environment using plants. Biotechnology.

[11] Nathanail C.P., Bardos R.P. (2004). Reclamation of Contaminated Land.

[12] Wan X., Lei M., Chen T. (2015). Cost–benefit calculation of phytoremediation technology for heavy-metal-contaminated soil. Sci. Total Environ..

[13] Baker A.J.M., Reeves R.D., McGrath S.P., Hinchee R.E., Olfenbuttel R.F. (1991). In situ decontamination of heavy metal polluted soils using crops of metal-accumulating plants—A feasibility study. In Situ Bioreclamation.

[14] Baker A.J.M., Mcgrath S.P., Sidoli C.M.D., Reeves R.D. (1994). The possibility of in situ heavy metal decontamination of polluted soils using crops of metal-accumulating plants. Resour. Conserv. Recycl..

[15] Clayton L. (2007). Phytoremediation. Encyclopedia of Plant and Crop Science.

[16] Koptsik G.N. (2014). Problems and prospects concerning the phytoremediation of heavy metal polluted soils: A review. Eurasian Soil Sci..

[17] Chen J., Chen Y., Shi Z.Q., Su Y., Han F.X., Ansari A., Gill S., Gill R., Lanza G., Newman L. (2015). Phytoremediation to remove metals/metalloids from soils. Phytoremediation: Management of Environmental Contaminants.

[18] Farraji H., Zaman N.Q., Tajuddin R.M., Faraji H. (2016). Advantages and disadvantages of phytoremediation: A concise review. Int. J. Environ. Tech. Sci..

[19] Syam N., Wardiyati T., Maghfoer M.D., Handayanto E., Ibrahim B., Muchdar A. (2016). Effect of Accumulator Plants on Growth and Nickel Accumulation of Soybean on Metal-contaminated Soil. Agric. Agric. Sci. Procedia.

[20] Eevers N., White J.C., Vangronsveld J., Weyens N., Cuypers A., Vangronsveld J. (2017). Bio- and Phytoremediation of Pesticide-Contaminated Environments: A Review. Advances in Botanical Research.

[21] Ernst W.H.O., Verkleij J.A.C., Schat H. (1992). Metal tolerance in plants. Acta Bot. Neerl..

[22] Kokyo O., Tiehua C., Tao L., Hongyan C. (2014). Study on Application of Phytoremediation Technology in Management and Remediation of Contaminated Soils. J. Clean Energy Technol..

[23] Sarma H. (2011). Metal hyperaccumulation in plants: A review focusing on phytoremediation technology. J. Environ. Sci. Technol..

[24] Bolan N.S., Adriano D.C., Curtin D. (2003). Soil acidification and liming interactions with nutrientand heavy metal transformationand bioavailability. Adv. Agron..

[25] Liu W., Zhou Q., An J., Sun Y., Liu R. (2010). Variations in cadmium accumulation among Chinese cabbage cultivars and screening for Cd-safe cultivars. J. Hazard. Mater..

[26] Paz-Alberto A.M., Sigua G.C. (2013). Phytoremediation: A Green Technology to Remove Environmental Pollutants. Am. J. Clim. Chang..

[27] Bhardwaj R., Sharma R., Handa N., Kaur H., Kaur R., Sirhindi G., Thukral A.K., Ahmad P., Saiema R. (2014). Prospects of Field Crops for Phytoremediation of Contaminants. Emerging Technologies and Management of Crop Stress Tolerance.

[28] Ali H., Khan E., Anwar Sajad M. (2013). Phytoremediation of heavy metals—Concepts and applications. Chemosphere.

[29] Alkorta I., Hernández-Allica J., Becerril J.M., Amezaga I., Albizu I., Onaindia M., Garbisu C. (2004). Chelate-enhanced phytoremediation of soils polluted with heavy metals. Rev. Environ. Sci. Biotechnol..

[30] Radziemska M. (2018). Study of applying naturally occurring mineral sorbents of Poland (dolomite halloysite, chalcedonite) for aided phytostabilization of soil polluted with heavy metals. Catena.

[31] Chaney R.L., Malik M., Li Y.M., Brown S.L., Brewer E.P., Angle J.S., Baker A.J.M. (1997). Phytoremediation of soil metals. Curr. Opin. Biotechnol..

[32] Brooks R.R. (1998). Plants That Hyperaccumulate Heavy Metals: Their Role in Phytoremediation, Microbiology, Archaeology, Mineral Exploration and Phytomining.

[33] Raskin I., Ensley B.D. (2000). Phytoremediation of Toxic Metals: Using Plants to Clean Up the Environment.

[34] Pulford I.D., Riddell-Black D., Stewart C. (2002). Heavy metal uptake by willow clones from sewage sludge-treated soil: The potential for phytoremediation. Int. J. Phytoremediat..

[35] Mendez M.O., Maier R.M. (2008). Phytostabilization of mine tailings in arid and semiarid environments—An emerging remediation technology. Environ. Health Perspect..

[36] Mattina M.J.I., Lannucci-Berger W., Musante C., White J.C. (2003). Concurrent plant uptake of heavy metals and persistent organic pollutants from soil. Environ. Pollut..

[37] Baker A.J.M., Brooks R.R. (1989). Terrestrial higher plants which hyperaccumulate metallic elements—A review of their distribution, ecology and phytochemistry. Biorecovery.

[38] Bedabati Chanu L., Gupta A. (2016). Phytoremediation of lead using Ipomoea aquatica Forsk. in hydroponic solution. Chemosphere.

[39] Armishaw R., Bardos R.P., Dunn R.M., Hill J.M., Pearl M., Rampling T., Wood P.A. (1992). Review of Innovative Contaminated Soil Clean-Up Processes.

[40] Radziemska M., Koda E., Bilgin A., Vaverková M.D. (2018). Concept of aided phytostabilization of contaminated soils in postindustrial areas. Int. J. Environ. Res. Public Health.

[41] Grobelak A., Napora A. (2015). The chemophytostabilisation process of heavy metal polluted soil. PLoS ONE.

[42] Stefaniuk M., Oleszczuk P., Ok Y.S. (2015). Review on nano zerovalent iron (nZVI): From synthesis to environmental applications. Chem. Eng. J..

[43] Xu Y., Liang X., Xu Y., Qin X., Huang Q., Wang L., Sun Y. (2017). Remediation of Heavy Metal-Polluted Agricultural Soils Using Clay Minerals: A Review. Pedosphere.

[44] Houben D., Pircar J., Sonnet P. (2012). Heavy metal immobilization by cost-effective amendments in a contaminated soil: Effects on metal leaching and phytoavailability. J. Geochem. Explor..

[45] Derakhshan Nejad Z., Jung M.C., Kim K.H. (2018). Remediation of soils contaminated with heavy metals with an emphasis on immobilization technology. Environ. Geochem. Health.

[46] Bryan G.W. (1980). Recent trends in research on heavy-metal contamination in the sea. Helgol. Meeresunters.

[47] Adiloğlu S., Turgut Sağlam M., Adiloğlu A., Süme A. (2016). Phytoremediation of nickel (Ni) from agricultural soils using canola (Brassica napus L.). Desalin. Water Treat..

[48] Bradl H. (2005). Heavy Metals in the Environment: Origin, Interaction and Remediation.

[49] Alloway B.J., Alloway B.J. (2013). Heavy Metals in Soils: Trace Metals and Metalloids in Soils and their Bioavailability.

[50] Spellman F.R. (2017). The Science of Environmental Pollution.

[51] (2006). Ministro Dell’ambiente e Della Tutela del Territorio Individuazione Dei Rifiuti non Pericolosi Sottoposti alle Procedure Semplificate di Recupero ai Sensi Degli Articoli 31 e 33 del Decreto Legislativo 5 Febbraio 1997, n. 22.

[52] Keller C., Hammer D., Kayser A., Richner W., Brodbeck M., Sennhauser M. (2003). Root development and heavy metal phytoextraction efficiency: Comparison of different plant species in the field. Plant Soil.

[53] Ciura J., Poniedziałek M., Sekara A., Jedrszczyk E. (2005). The possibility of using crops as metal phytoremediants. Pol. J. Environ. Stud..

[54] Meers E., Ruttens A., Hopgood M., Lesage E., Tack F.M.G. (2005). Potential of Brassic rapa, Cannabis sativa, Helianthus annuus and Zea mays for phytoextraction of heavy metals from calcareous dredged sediment derived soils. Chemosphere.

[55] Revathi K., Haribabu T.E., Sudha P.N. (2011). Phytoremediation of Chromium contaminated soil using Sorghum plant. Int. J. Environ. Sci..

[56] Wilson-Corral V., Anderson C., Rodriguez-Lopez M., Arenas-Vargas M., Lopez-Perez J. (2011). Phytoextraction of gold and copper from mine tailings with Helianthus annuus L. and Kalanchoe serrata L.. Miner. Eng..

[57] Gunduz S., Uygur F.N., Kahramano İ. (2012). Heavy Metal Phytoremediation Potentials of Lepidum sativum L., Lactuca sativa L., Spinacia oleracea L. and Raphanus sativus L.. Her. J. Agric. Food Sci. Res..

[58] Chaturvedi R., Favas P.J., Pratas J., Varun M., Paul M.S. (2019). Metal(loid) induced toxicity and defense mechanisms in Spinacia oleracea L.: Ecological hazard and Prospects for phytoremediation. Ecotoxicol. Environ. Saf..

[59] Giordani C., Cecchi S., Zanchi C. (2005). Phytoremediation of soil polluted by nickel using agricultural crops. Environ. Manag..

[60] Solhi M., Hajabbasi M.A., Shareatmadari H. (2005). Heavy Metals Extraction Potential of Sunflower (Helianthus annuus) and Canola (Brassica napus). Casp. J. Environ. Sci..

[61] Turan M., Esringü A. (2007). Phytoremediation based on canola (Brassica napus L.) and Indian mustard (Brassica juncea L.) planted on spiked soil by aliquot amount of Cd, Cu, Pb, and Zn. Plant Soil Environ..

[62] Van Ginneken L., Meers E., Guisson R., Ruttens A., Elst K., Tack F.M.G., Vangronsveld J., Diels L., Dejonghe W. (2007). Phytoremediation for heavy metal-contaminated soils combined with bioenergy production. J. Environ. Eng. Landsc. Manag..

[63] Dell’Amico E., Cavalca L., Andreoni V. (2008). Improvement of Brassica napus growth under cadmium stress by cadmium-resistant rhizobacteria. Soil Biol. Biochem..

[64] Verdiloo H.K., Homaee M. (2008). Modeling of cadmium and lead phytoextraction from contaminated soils. Pol. J. Soil Sci..

[65] Zhuang P., Shu W., Li Z., Liao B., Li J., Shao J. (2009). Removal of metals by sorghum plants from contaminated land. J. Environ. Sci..

[66] Adesodun J.K., Atayese M.O., Agbaje T.A., Osadiaye B.A., Mafe O.F., Soretire A.A. (2010). Phytoremediation potentials of sunflowers (Tithonia diversifolia and Helianthus annuus) for metals in soils contaminated with zinc and lead nitrates. Water Air Soil Pollut..

[67] Casucci C., De Bernardi A., D’Amato R., Businelli D., Vischetti C. (2020). Zeolite and bentonite as nickel sequestrants in carbonation lime coming from the sugar industry. Environ. Sci. Pollut. Res..

[68] Marchiol L., Sacco P., Assolari S., Zerbi G. (2004). Reclamation of polluted soil: Phytoremediation potential of crop-related Brassica species. Water. Air Soil Pollut..

[69] Purakayastha T.J., Viswanath T., Bhadraray S., Chhonkar P.K., Adhikari P.P., Suribabu K. (2008). Phytoextraction of zinc, copper, nickel and lead from a contaminated soil by different species of Brassica. Int. J. Phytoremediat..

[70] Çakmakci T., Ucar Y. (2014). Efficiency of canola (Brassica Napus L.) as an accumulator of heavy metals in wastewater applications. Pol. J. Environ. Stud..

[71] Salaskar D., Shrivastava M., Kale S.P. (2011). Bioremediation potential of spinach (Spinacia oleracea L.) for decontamination of cadmium in soil. Curr. Sci..

[72] Pathak C., Chopra A.K., Srivastava S. (2013). Accumulation of heavy metals in Spinacia oleracea irrigated with paper mill effluent and sewage. Environ. Monit. Assess..

[73] Alia N., Sardar K., Said M., Salma K., Sadia A., Sadaf S., Toqeer A., Miklas S. (2015). Toxicity and bioaccumulation of heavy metals in spinach (Spinacia oleracea) grown in a controlled environment. Int. J. Environ. Res. Public Health.

[74] Ling W., Shen Q., Gao Y., Gu X., Yang Z. (2007). Use of bentonite to control the release of copper from contaminated soils. Aust. J. Soil Res..

[75] Akpomie K.G., Dawodu F.A. (2015). Potential of a low-cost bentonite for heavy metal abstraction from binary component system. Beni-Suef Univ. J. Basic Appl. Sci..

[76] Chaves L.H.G., Tito G.A., De Brito Chaves I. (2017). Bentonite application in the remediation of zinc contamination soil. Chem. Eng. Trans..

[77] Brunetti G., Farrag K., Soler-Rovira P., Nigro F., Senesi N. (2011). Greenhouse and field studies on Cr, Cu, Pb and Zn phytoextraction by Brassica napus from contaminated soils in the Apulia region, Southern Italy. Geoderma.

[78] Panwar B.S., Ahmed K.S., Mittal S.B. (2001). Phytoremediation of nickel-contaminated soils by Brassica species. Environ. Dev. Sustain..

[79] Farraji H., Aziz H.A., Tajuddin R.M., Mojiri A. (2014). Optimization of Phytoremediation of Lead-contaminated Soil by Spinach (Spinacia oleracea L). Int. J. Sci. Res. Knowl..

[80] Nachtegaal M., Sparks D.L. (2003). Nickel sequestration in a kaolinite-humic acid complex. Environ. Sci. Technol..

[81] Wasilkowski D., Nowak A., Płaza G., Mrozik A. (2017). Effects of pulp and Na-bentonite amendments on the mobility of trace elements, soil enzymes activity and microbial parameters under ex situ aided phytostabilization. PLoS ONE.

[82] Marchiol L., Fellet G., Perosa D., Zerbi G. (2007). Removal of trace metals by Sorghum bicolor and Helianthus annuus in a site polluted by industrial wastes: A field experience. Plant Physiol. Biochem..

[83] Luo Q., Sun L., Hu X., Zhou R. (2014). The variation of root exudates from the hyperaccumulator Sedum alfredii under cadmium stress: Metabonomics analysis. PLoS ONE.

[84] Montiel-Rozas M.M., Madejón E., Madejón P. (2016). Effect of heavy metals and organic matter on root exudates (low molecular weight organic acids) of herbaceous species: An assessment in sand and soil conditions under different levels of contamination. Environ. Pollut..

[85] Chen Y.T., Wang Y., Yeh K.C. (2017). Role of root exudates in metal acquisition and tolerance. Curr. Opin. Plant Biol..

[86] Soudek P., Petrová Š., Vaňková R., Song J., Vaněk T. (2014). Accumulation of heavy metals using Sorghum sp.. Chemosphere.

[87] Angelova V.R., Ivanova R.V., Delibaltova V.A., Ivanov K.I. (2011). Use of Sorghum Crops for in Situ Phytoremediation of Polluted Soils. J. Agric. Sci. Technol..

[88] Naeini J., Yousefi Rad M., Rad M.Y. (2018). Phytoremediation capability of nickel and manganese polluted soil by Sorghum biocilor L.. Plant Physiol..

[89] Walkley A., Black I.A. (1934). An examination of the degtjareff method for determining soil organic matter, and a proposed modification of the chromic acid titration method. Soil Sci..

[90] (2013). Ente Nazionale Italiano di Unificazione Rifiuti-Campionamento Manuale, Preparazione del Campione ed Analisi Degli Eluati.

[91] (1999). Ministero Delle Politiche Agricole e Forestali D.M. Approvazione dei “Metodi Ufficiali di Analisi Chimica del Suolo”.

[92] Huang L., Bell R.W., Dell B., Woodward J. (2004). Rapid Nitric Acid Digestion of Plant Material with an Open-Vessel Microwave System. Commun. Soil Sci. Plant Anal..

[93] Bender R., Lange S. (2001). Adjusting for multiple testing—When and how?. J. Clin. Epidemiol..

[94] Chen S.Y., Feng Z., Yi X. (2017). A general introduction to adjustment for multiple comparisons. J. Thorac. Dis..

[95] Sengar R.S., Gupta S., Gautam M., Sharma A., Sengar K. (2008). Occurrence, uptake, accumulation and physiological responses of Nickel in plants and its effects on environment. Res. J. Phytochem..

[96] Arnao M.B., Hernández-Ruiz J. (2019). Role of Melatonin to Enhance Phytoremediation Capacity. Appl. Sci..

[97] Asif M., Pervez A., Ahmad R. (2019). Role of Melatonin and Plant-Growth-Promoting Rhizobacteria in the Growth and Development of Plants. Clean Soil Air Water.

[98] Seleiman M.F., Ali S., Refay Y., Rizwan M., Alhammad B.A., El-Hendawy S.E. (2020). Chromium resistant microbes and melatonin reduced Cr uptake and toxicity, improved physio-biochemical traits and yield of wheat in contaminated soil. Chemosphere.

