# Supplementary material for: Phytoremediation Potential of Crop Plants in Countering Nickel Contamination in Carbonation Lime Coming from the Sugar Industry
Source: Plants (Basel). 2020 May 2;9(5):580. doi: 10.3390/plants9050580 (PMC7285043; doi:10.3390/plants9050580)
Supplement: Supplementary file 1 [file plants-09-00580-s001.pdf]

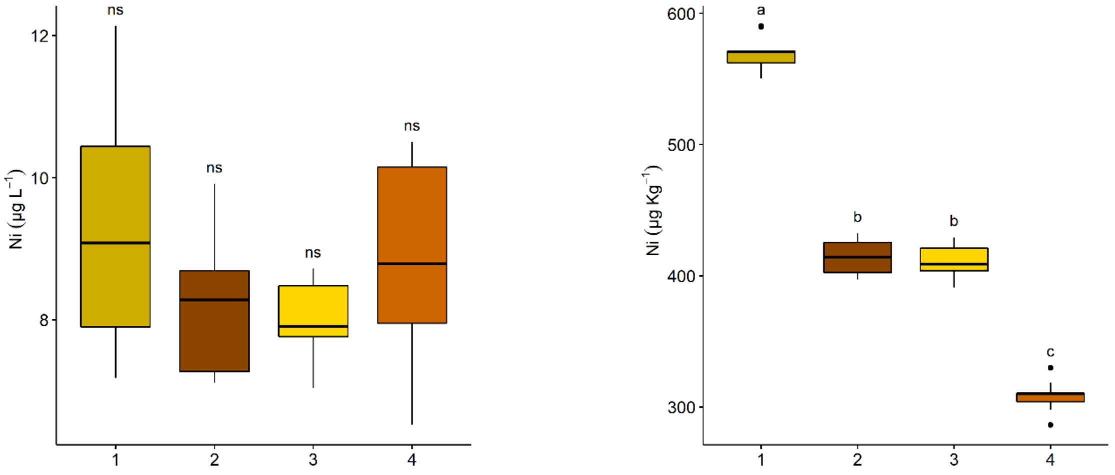

(a) (b)

Figure S1. (**a**) Pre-sowing concentration of soluble Ni (µg L^{-1}) in lime within boxes 1-4; (**b**) Pre-sowing concentration of biovaiable Ni (µg Kg^{-1}) in lime within boxes 1-4. Number in the x-axis refer to the four boxes. Dunn's Kruskal-Wallis multiple comparisons (Benjamini-Hochberg p-value adjustment, α-level=0.05).

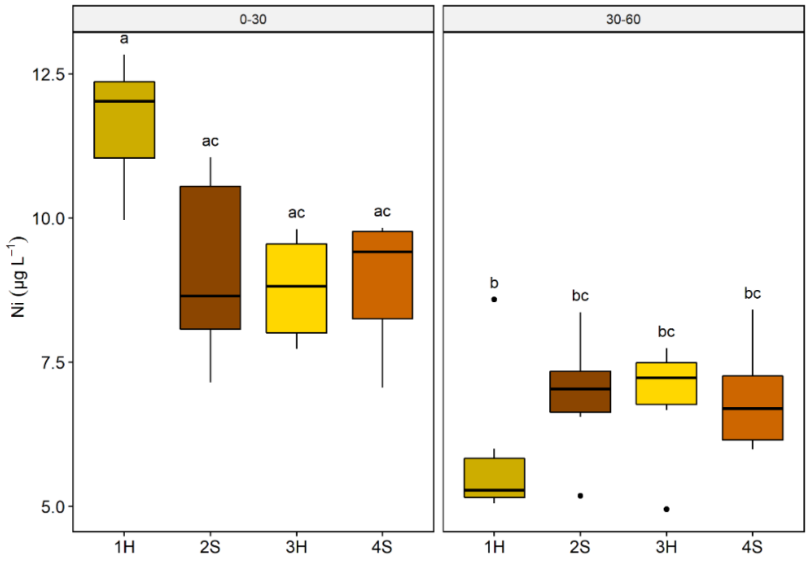

(a)

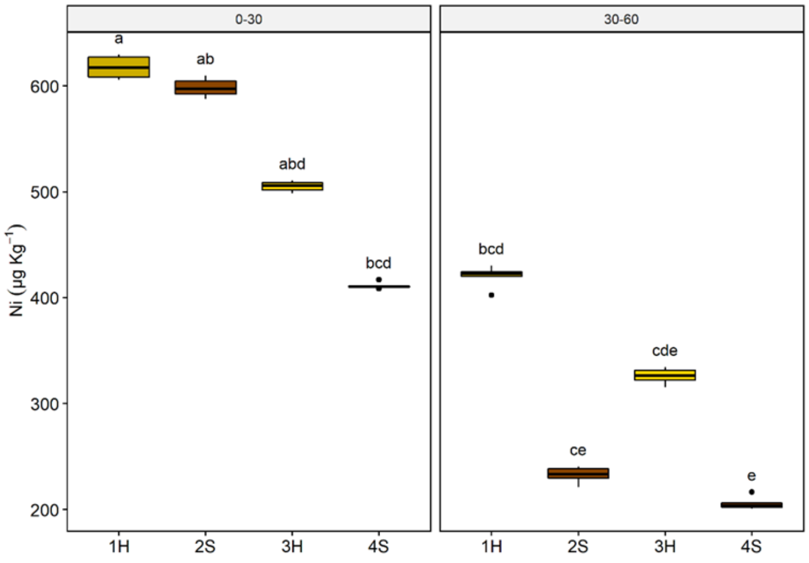

(b)

Figure S2. (a) Post-harvest concentration of soluble Ni (µg L^{-1}) in lime within boxes 1-4. (b) Post-harvest concentration of bioavailable Ni (µg Kg^{-1}) in lime within boxes 1-4. Box 1 and 3 after *Helianthus annuus* (1H, 3H); box 2 and 4 after *Sorghum vulgare* (2S, 4S). Ni concentration was grouped by depth of sampling (0-30 cm, 30-60 cm). Dunn's Kruskal-Wallis multiple comparisons (Benjamini-Hochberg p-value adjustment, α-level=0.05) was performed using data from both sampling depths.

Table S1. Dry weight (g) of biomass per plant in the greenhouse experiment (reported values are mean ± standard deviation).

Trial	Hypogeal	Epigeal
B	4.52±0.64	15.16±0.87
B+	4.40±0.81	14.71±1.22
BC	4.50±0.80	14.58±1.07
S	0.79±0.11	5.91±0.39
S+	0.37±0.10	4.73±0.51
SC	0.56±0.14	5.97±0.49

Table S2. Dry weight (g) of biomass per plant in the outdoor experiment (reported values are mean ± standard deviation).

Box	Root	Stem	Leaf	Infructescence
1H	22.37±2.38	81.16±3.89	27.30±2.77	33.58±2.51
3H	27.21±2.19	87.66±3.27	44.50±3.72	37.00±2.27
H	27.09±1.89	88.43±2.32	41.17±1.67	37.00±3.53
2S	14.59±1.19	38.86±1.87	20.76±1.50	13.98±2.13
4S	16.98±2.00	39.54±1.46	21.60±2.20	14.19±2.43
S	15.66±2.20	37.32±2.66	21.75±2.87	14.13±3.00
